# Evaluation of a multibody kinematics optimization method for three-dimensional canine pelvic limb gait analysis

**DOI:** 10.1186/s12917-020-02323-5

**Published:** 2020-04-03

**Authors:** Cheng-Chung Lin, Ching-Ho Wu, Po-Yen Chou, Shi-Nuan Wang, Wei-Ru Hsu, Tung-Wu Lu

**Affiliations:** 1 Department of Electrical Engineering, Fu Jen Catholic University, New Taipei City, Taiwan; 2 Institute of Veterinary Clinical Science, School of Veterinary Medicine, National Taiwan University, Taipei, Taiwan; 3 Department of Surgical and Radiological Science, School of Veterinary Medicine, University of California Davis, Davis, CA USA; 4 Department of Biomedical Engineering and Department of Orthopedic Surgery, School of Medicine, National Taiwan University, Taipei, Taiwan

**Keywords:** Fluoroscopy, Gait analysis, Kinematics, Locomotion, Multibody kinematics optimization, Soft tissue artefact

## Abstract

**Background:**

Skin marker-based three-dimensional kinematic gait analysis were commonly used to assess the functional performance and movement biomechanics of the pelvic limb in dogs. Unfortunately, soft tissue artefact would compromise the accuracy of the reproduced pelvic limb kinematics. Multibody kinematics optimization framework was often employed to compensate the soft tissue artefact for a more accurate description of human joint kinematics, but its performance on the determination of canine pelvic limb skeletal kinematics has never been evaluated. This study aimed to evaluate a multibody kinematics optimization framework used for the determination of canine pelvic limb kinematics during gait by comparing its results to those obtained using computed tomography model-based fluoroscopy analysis.

**Results:**

Eight clinically normal dogs were enrolled in the study. Fluoroscopy videos of the stifle joint and skin marker trajectories were acquired when the dogs walked on a treadmill. The pelvic limb kinematics were reconstructed through marker-based multibody kinematics optimization and single-body optimization. The reference kinematics data were derived via a model-based fluoroscopy analysis. The use of multibody kinematics optimization yielded a significantly more accurate estimation of flexion/extension of the hip and stifle joints than the use of single-body optimization. The accuracy of the joint model parameters and the weightings to individual markers both influenced the soft tissue artefact compensation capability.

**Conclusions:**

Multibody kinematics optimization designated for soft tissue artefact compensation was established and evaluated for its performance on canine gait analysis, which provided a further step in more accurately describing sagittal plane kinematics of the hip and stifle joints.

## Background

The clinical assessment of canine lameness typically relies on qualitative examination by veterinarians, yet subtle changes in functional performance are difficult to detect without quantitative information. Kinematics analysis of canine gait has therefore increasingly been used to quantify breed specific biomechanical characteristics [1, 2], to explore the pathomechanics in relation to orthopedic disorders [3, 4], and to evaluate treatment outcomes [5, 6]. While several methodologies and equipment have been developed for kinematic analysis in canine patients, [2, 7–9], skin marker-based motion analysis and computed tomography (CT) model-based fluoroscopic analysis are the most frequently used approaches in canine gait assessment [2, 10, 11].

The estimation of joint kinematics using skin marker-based motion analysis requires a motion capture system to capture the skin marker trajectories during motion. The body segment of interest is then subsequently analysed using an appropriate kinematic model. Sagittal plane kinematics of the canine stifle have been reported in a two-dimensional (2-D) linkage model using joint rotation centres specified by skin markers [12]. In addition, comprehensive assessments of joint kinematics in the sagittal, frontal and transverse planes have been reported in three-dimensional (3-D) kinematic models [6, 13, 14].

The deformation and displacement of the skin creates soft tissue artefact (STA) [15], which result in femoral and tibial length changes and deviated angle estimations throughout the gait cycle [16, 17]. Compensation of STA via rigidifying the segment models was first reported by Kim et al. in a 2-D linkage model [16] and by Fu and Torres et al. in a 3-D segment model [13, 14]. In 3-D gait analysis, the process of rigidifying an individual segment model is also called the single-body optimization (SO) strategy or segmental optimization. Single-body optimization estimates the corresponding segment by minimizing the deformation of the measured marker array from its initial shape [14, 18], addressing the nonrigid error component of the STA. However, SO cannot correct the STA error composited by rigid movement components [19], and residual errors in stifle angle estimation have been observed in dogs [15, 20]. While other strategies, such as traditional filtering, dynamic calibration and point cluster techniques, have been reported in human motion analysis to compensate for the STA [21], they either were not able to accurately estimate 3-D knee kinematics or may not be feasible for dogs.

Multibody kinematics optimization (MKO) utilizes the overall pelvic limb model and imposes joint constraints to connect the adjacent segments [22]. MKO was introduced to provide another STA-compensated solution for the estimation of 3-D kinematics of the pelvic limb by minimizing the differences between model-determined and measured skin marker coordinates using a global optimization strategy [22, 23]. The MKO approach and its modifications have been widely assessed for their STA compensation capability in human gait analysis [23–28]. MKO offers the advantage that it can be implemented with conventional 3-D marker sets and therefore could provide a possible alternative for STA compensation in canine motion analysis. While MKO has been reported to be available for canine kinematics analysis after model modification for accommodating the dog conformation [9], its performance in the determination of STA-free joint kinematics has never been evaluated.

Computed tomography model-based fluoroscopic analysis has also been reported for estimating STA-free segment and joint kinematics in dogs [2, 8, 11, 15]. It integrates CT-based models and fluoroscopy images [29] and can serve as the reference standard for evaluating other motion measurements [15, 20, 30]. While CT model-based fluoroscopic analysis is advantageous in obtaining a relatively accurate spatial pose of the body segments (accuracy: 0.77 mm and 3.06 mm for translations and 1.13° for rotations [29]), its major disadvantages are radiation exposure, the need for CT scanning and reconstruction, additional costs, and the narrow field of view of fluoroscopy imaging, which limits the motion measurement to a single joint (or 2–3 adjacent body segments). Using an STA compensation strategy to improve the accuracy of marker-based motion analysis to a level close to that of CT model-based fluoroscopic analysis is still warranted to facilitate canine kinematics measurement with fewer experimental constraints.

The study aimed to develop an MKO framework for dogs that allow the determination of model parameters directly from markers and to evaluate the errors of the MKO-determined hip and stifle joint kinematics of canine gait. The influence of model parameters regarding the existence of joint constraints, the accuracy of joint centre locations and the weighting factors were analysed. We hypothesize that compared to SO, MKO, when used in marker-based motion analysis, would lead to a more accurate reproduction of joint kinematics.

## Results

**Fig. 1:**
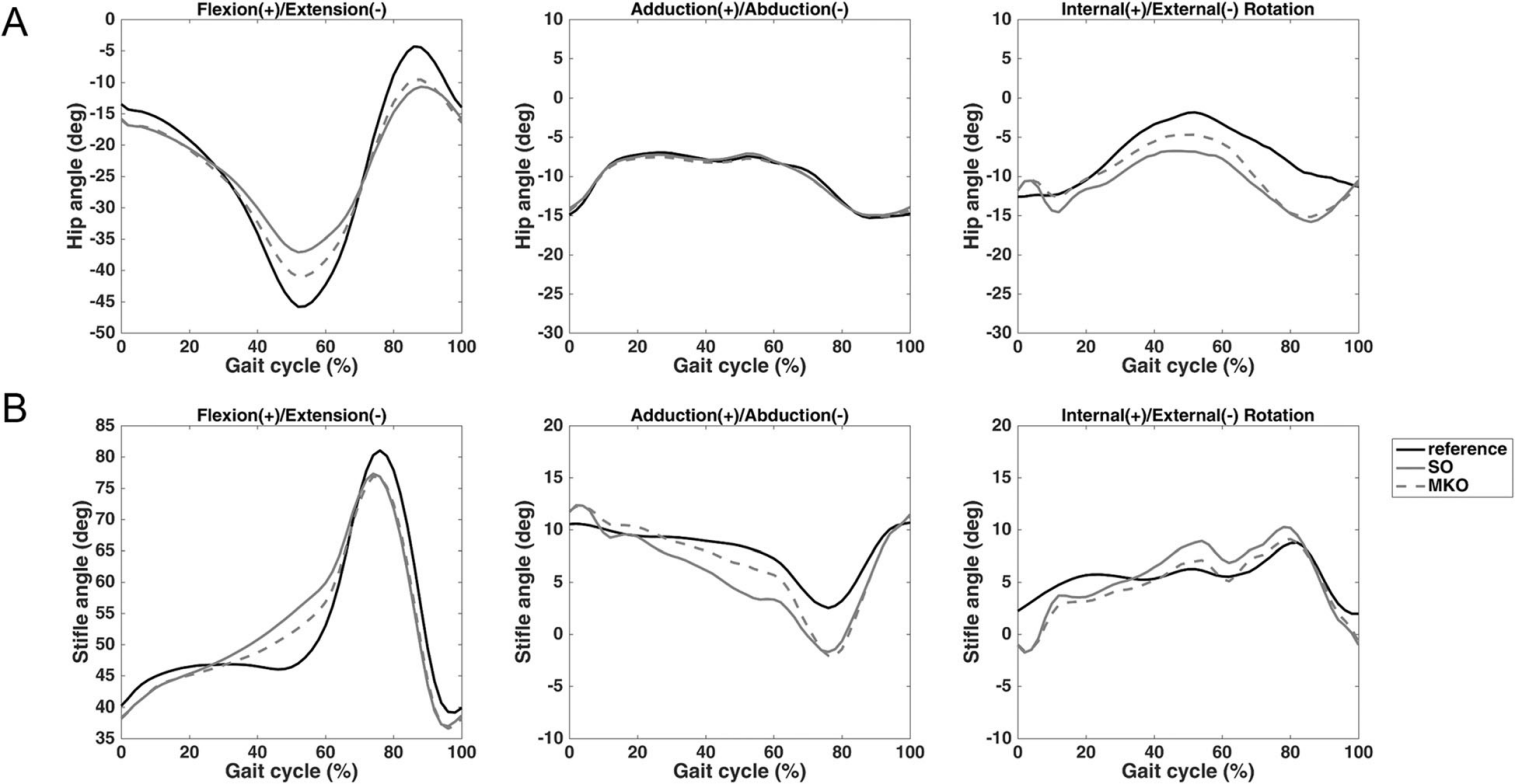
Waveforms of the hip and stifle joint angles. **a** Hip joint angles and **b** stifle joint angles in three anatomical planes obtained using the model-based fluoroscopy analysis (reference), SO and MKO methods

**Fig. 2:**
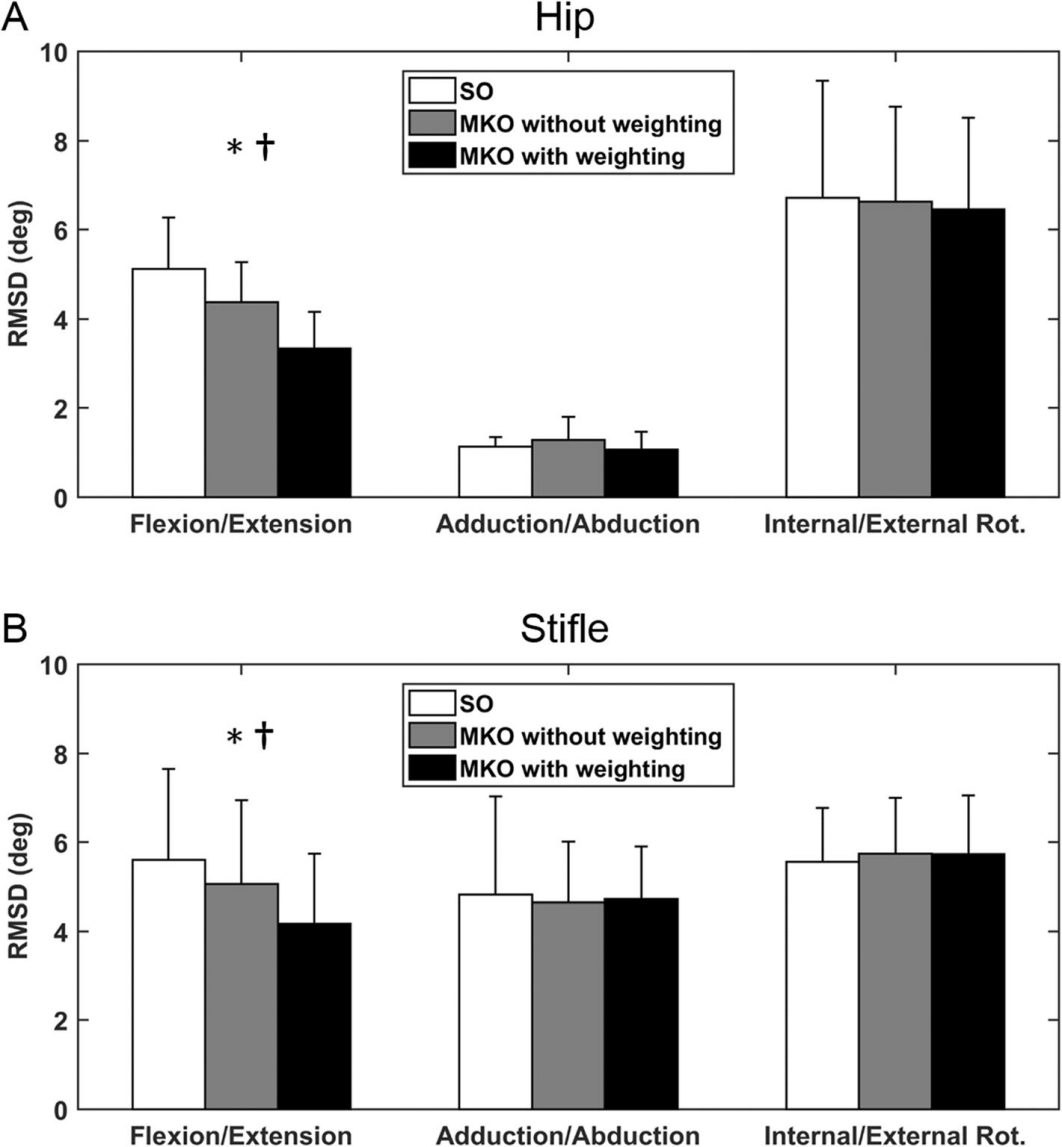
Comparison of the RMSD between SO and MKO methods. **a** The RMSD of the hip joint angles obtained using SO and MKO with and without specified weightings. **b** The corresponding values for the stifle joint. The asterisk indicates the significant differences in RMSD between SO and MKO without weighting methods, while the dagger shows the significance between SO and MKO with weighting methods

The averaged waveforms of the hip and stifle angles in the sagittal, transverse and frontal planes during a gait cycle obtained using MKO and SO and from reference values are presented in Fig. 1. Quantitatively, higher bias and lower confidence interval (CI) values were obtained using MKO than using SO for both joints in sagittal plane motion (Table 1). However, no considerable differences were found in other motion components. Similar results were also found in the root mean square difference (RMSD), an index used to account for the overall differences between the MKO (or SO)-determined kinematic waveforms and the reference waveforms. The greater the RMSD, the higher the discrepancy between the waveforms. The use of MKO yielded a significantly more accurate estimation of flexion/extension of the hip and stifle joints than the SO method (Fig. 2).

**Table 1:** Degree of agreement between joint angles derived from the SO and MKO procedures and reference joint angles

Model	Weighting	Hip Flexion/Extension			Hip Adduction/Abduction			Hip Internal/External Rotation		
		bias	CI	R^2^	bias	CI	R^2^	bias	CI	R^2^
SO	No	−0.1	10.2	0.908	0.1	2.2	0.965	−2.6	12.8	0.633
MKO	No	−0.5	8.6	0.948	0.2	2.7	0.956	−2.0	12.9	0.642
MKO	Yes	−0.9	6.5	0.977	−0.1	2.2	0.966	−1.6	12.8	0.633
		Stifle Flexion/Extension			Stifle Adduction/Abduction			Stifle Internal/External Rotation		
		bias	CI	R^2^	bias	CI	R^2^	bias	CI	R^2^
SO	No	−0.1	11.5	0.825	−1.5	9.7	0.553	−0.7	11.1	0.741
MKO	No	−0.4	10.4	0.855	−1.0	9.2	0.609	−0.9	11.4	0.720
MKO	Yes	−0.9	8.4	0.904	−0.6	9.4	0.600	−1.4	11.3	0.733

The bias and CI values were determined with Bland-Altman analysis. The coefficient of determination values (R^2^) were obtained from linear regression analysis. The units for bias and CI are degrees

**Fig. 3:**
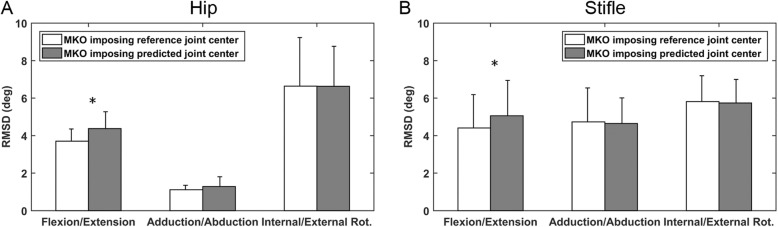
Comparison of the RMSD between MKO imposing reference and predicted joint centres. The RMSD obtained using MKO imposing reference joint centres and predicted joint centres for the determination of flexion/extension, adduction/abduction, and internal/external rotation of (**a**) hip and (**b**) stifle joints. The asterisk indicates the significant difference of RMSD obtained with the two methods

**Fig. 4:**
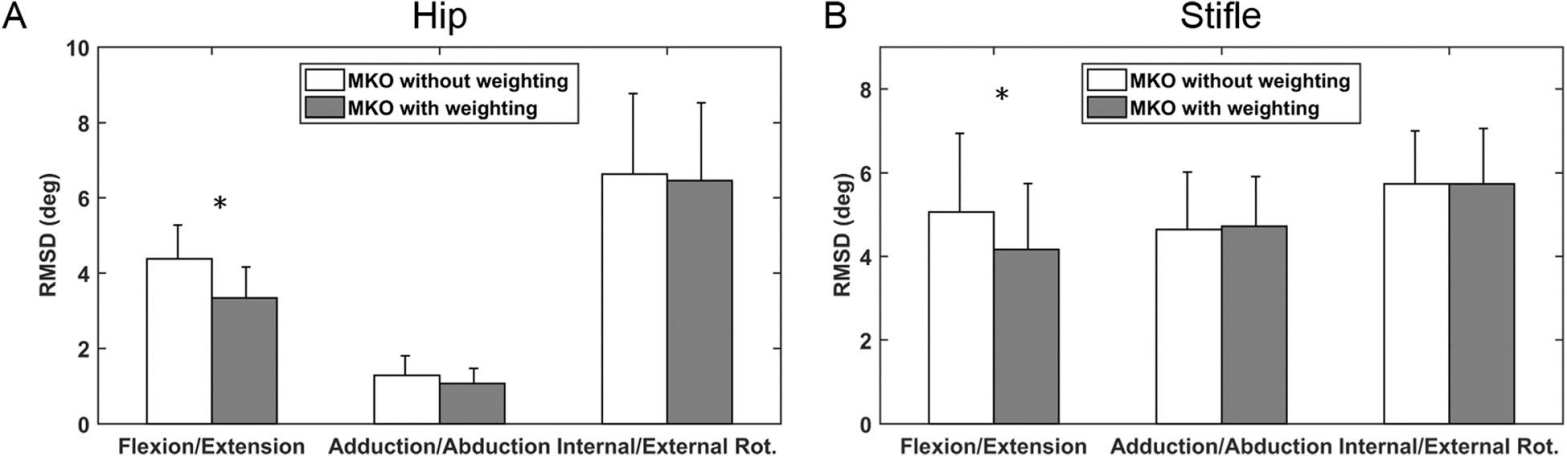
Comparison of the RMSD between MKO with and without weightings. The RMSD obtained using MKO with and without specified weightings for the determination of flexion/extension, adduction/abduction, and internal/external rotation of (**a**) hip and (**b**) stifle joints. The asterisk indicates the significant difference in RMSD obtained with the two methods

The hip joint centre (HJC) position predicted based on anatomical landmarks was significantly different from the reference joint centre position in the cranial/caudal and proximal/distal directions, in which their total difference was 16.7 mm (Table 2). While no significant differences were found between the predicted and reference stifle joint centres (SJCs), the error magnitude also achieved 7.5 mm (Table 2). Given the discrepancy in the joint centre positions, the MKO imposing predicted and reference joint centres were also found to yield significantly discrepant estimations of flexion/extension of the hip and stifle joints (Fig. 3). In addition, MKO with specified weightings to individual markers gave a more accurate estimation of the flexion/extension angle with significantly lower RMSDs compared to that without weightings (Fig. 4).

**Table 2:** Comparison between reference and predicted joint centres

Kinematic Constraints		Cranial/Caudal		Proximal/Distal		Lateral/Medial		Total Distance Error
		mean ± SD (mm)	*p*-value	mean ± SD (mm)	*p*-value	mean ± SD (mm)	*p*-value	mean ± SD (mm)
Hip joint centre	Reference	− 83.6 ± 15.2	0.034	−36.9 ± 3.4	0.016	− 11.5 ± 10.9	0.547	16.7 ± 5.4
	Prediction	−89.8 ± 17.2		−25.3 ± 8.3		−9.7 ± 12.9		
Stifle joint centre	Reference	0.7 ± 6.1	0.743	−1.2 ± 5.5	0.543	1.3 ± 2.0	0.109	7.5 ± 3.5
	Prediction	0.0 ± 0.0		0.0 ± 0.0		0.0 ± 0.0		

The reference and predicted 3-D positions (in mm) of the hip and stifle joint centres in corresponding AFs. The total distance errors between the reference and predicted joint centres are also presented. Statistical comparisons were made with a significance level of 0.05

## Discussion

Overall, while MKO with kinematics constraint on joint centres could not provide an accuracy comparable to the model-based fluoroscopic analysis as indicated by the bias, CI and RMSD (Table 1 and Fig. 2), the > 0.9 determination coefficients observed with flexion/extension and adduction/abduction of the hip and with flexion/extension of the stifle joint support the usefulness of MKO. When comparing the MKO- and SO-determined kinematic waveforms (Fig. 1) and the resulting RMSDs (Fig. 2), the MKO method outperformed the SO method in estimating the flexion/extension angles of the hip and knee joints. It appeared that the use of the multibody model with spherical constraints was effective in partially compensating STA influence on the flexion/extension angle estimation regardless of whether the weighting factors were applied. In contrast, this model was not helpful for other kinematic components and sometimes even led to a more inaccurate result (e.g., stifle internal/external rotation). This may be explained by the combined effects of the STA and joint model imperfections [26] resulting from simplified joint geometry and joint centre position errors.

The reason for selecting the spherical joint model in the current multibody model is twofold. As indicated in a previous report on human motion [25], it was suggested that spherical constraint is a reasonable approximation when the knee joint is under limited flexion and small displacement, corresponding to the case of canine gait during which the range of stifle flexion is only 40° [11]. A more physiological representation of the stifle joint is intuitively a better choice for physiological description of joint kinematics such as articular linear displacements and contact patterns [31]. However, except for spherical and hinge type models, other existing joint model designs require precise geometry of bone, articulation, and ligaments [24, 25, 28]. In addition, a previous report suggested that the best joint model type may be motor task-dependent [27]. A thorough assessment of MKO embedding more complex joint models is warranted for a better description of pelvic limb kinematics during canine gait.

**Fig. 5:**
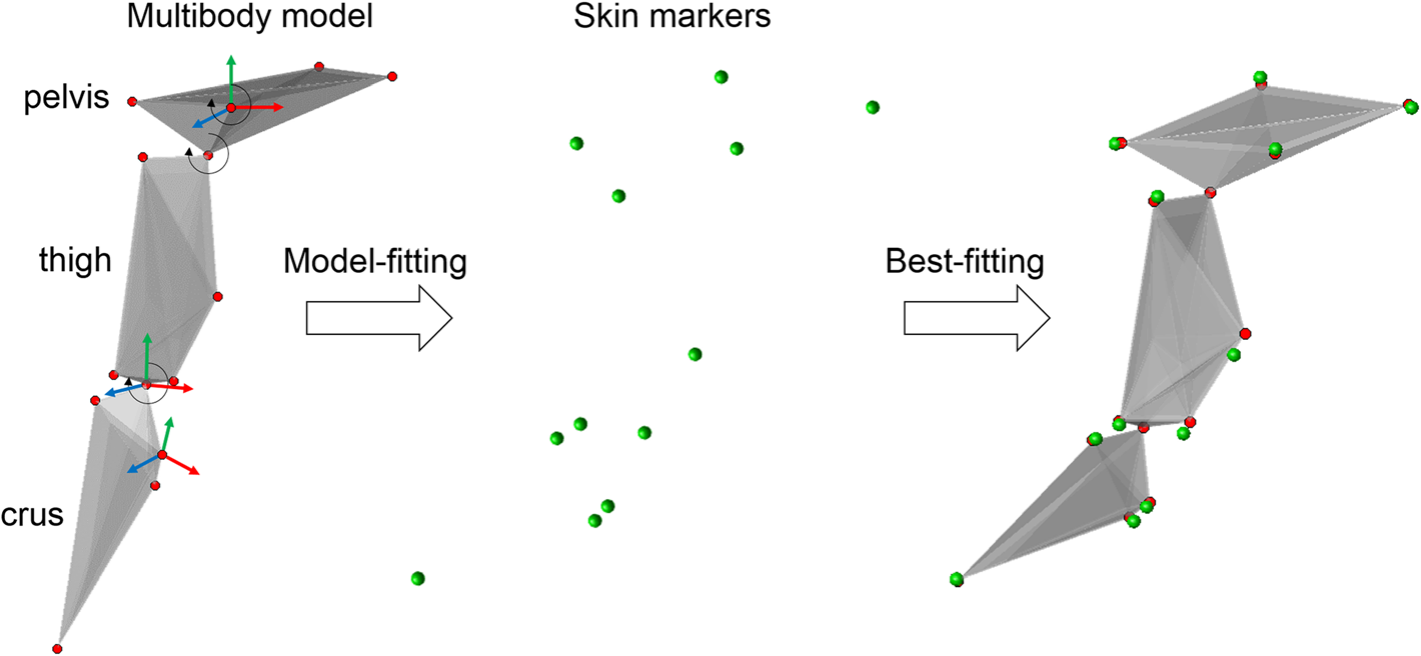
Procedure of the multibody kinematics model fitted to the skin markers. The multibody model is composed of pelvic, thigh and crus segments with hip and stifle joint centres connecting the adjacent segments (left). The multibody kinematics optimization was carried out to best fit the local markers on the model construct to the measured skin markers (middle and right) by finding the optimal set of model pose parameters

In the current study, landmarks of the femoral epicondyle provided a satisfactory estimation of the geometrical SJC, while the regression model-derived HJC achieved an error up to 1.6 cm (Table 2). In addition, it was also shown that the use of a multibody model with a set of joint centres closer to the true anatomical features helped improve the estimation of the joint flexion/extension angles with the MKO method (Fig. 3). The incorrect geometry of the multibody model caused by the biased HJC position may have directly contributed to the additional artificial differences between the model-determined and measured marker positions during the MKO analysis (Fig. 5) in addition to the STA-induced errors, compromising the performance of the MKO. More precise model parameters, obtained either from functional calibration [32] or radiography images [9, 28], contributing to subject-specific customization (or personalization) of multibody models may improve the STA compensation capability [23].

Assigning specific weightings to the markers also appeared to be effective for improving the performance of MKO, which led to a more accurate estimation of hip and stifle flexion/extension (Fig. 4). However, the influences on the other two motion components were marginal. In the current study, weightings were specified based on the local marker displacements, which were intended to mimic the status of the regional STAs. However, this approach is considered suboptimal since local marker displacements are derived based on the estimated body segment poses using the SO. The SO-determined segment poses are indeed subject to a rigid error component of STAs [20], which leads the local marker displacements to substantially underestimate the magnitude of STAs. Nonetheless, the computed weightings of the pelvis, thigh, and crus seem to follow a tendency that the thigh marker should be assigned lower weightings owing to greater STAs [15].

In the current study, the reference hip joint angle was not completely STA-free because the pelvic poses were determined via markers. This may have made the derived reference angles closer to the fully marker-based measurements (i.e., the MKO- and SO-determined hip joint angles), probably affecting the magnitudes of the errors of the MKO- and SO-determined hip joint angles. Nonetheless, the influences on the comparisons between the models (MKO vs. SO) and parameters (joint centres and weightings) were expected to be marginal since the partially STA-affected hip joint angle was the common reference for error evaluation. A separate X-ray imaging analysis of the hip joint during the canine gait in future studies may help justify the inference and further delineate the STA from the pelvic markers. Simplified stifle spherical models embedded in MKO yield nonphysiological stifle kinematics (e.g., null articular translations) and cannot provide fully reliable kinematics estimation. Further modifications of MKO, especially in the more complex stifle joint model and by taking genuine STA patterns into account, may help address these issues. Moreover, the study was limited in the low number of gait cycles (*n* = 3) chosen for the kinematics analysis. This is primarily due to the restricted field of view of X-ray imaging such that in most motion trials, the stifle would be out-of-view in a number of image frames during a gait cycle. Upgrading to a larger fluoroscopy image intensifier or finding a way to more precisely control the gait speed of the dogs may help improve the efficiency of data acquisition in canine gait analysis in future studies.

## Conclusions

In conclusion, this study quantitatively assessed the performance of MKO for the measurement of pelvic limb kinematics during canine locomotion. Promising results for the use of MKO in STA compensation of hip and stifle flexion/extension angles were found. Accurate joint geometrical parameters and appropriate weightings appeared to be crucial factors that affected the performance of MKO.

## Methods

### Marker sets

After shaving the hair around the locations of markers, the required skin markers were affixed to the skin surfaces of the pelvis and right hindlimb with double-sided tape and cyanoacrylate while the dogs stood still on the ground. Twelve anatomical landmark markers and two tracking markers were attached to designated locations on the subject’s pelvic limb. The 12 anatomical landmarks for marker placement were the right and left iliac crests, the right and left ischial tuberosities, the greater trochanter (GT), the lateral and medial femoral epicondyles, the fibular head, the proximal and distal tibial crests, and the lateral and medial malleoli. Two tracking markers were attached to the cranial aspect of the thigh to ensure that the 4-marker convention was not violated [33], as the medial side markers were eliminated during subsequent motion data acquisition [15]. For the construction of the subject-specific multibody model, a subject calibration was carried out to acquire coordinates of all the markers when the dog stood still on the ground. One additional tracking marker was attached to the dorsal aspect of the foot to define the time of paw contact for a complete gait cycle.

### Multibody kinematics optimization

The multibody model of the pelvic limb was composed of three rigid segments (pelvis, femur, and tibia) and two joint kinematic constraints (hip and stifle joints) (Fig. 5). The hip and stifle kinematic constraints were modeled as spherical joints (ball-and-socket). The multibody model was customized to individual dogs via the skin markers obtained from subject calibration. Each segment was imbedded with an anatomical frame (AF) given the corresponding marker array (Fig. 5), for which the pelvic and tibial AFs were determined following the reported recommendation [14], and the femoral AF was constructed in reference to the definition proposed by Cappozzo et al. [34]. The position vectors of the markers were then expressed in their corresponding AFs to form “marker templates” of the pelvic, femoral and tibial segments [20].

The HJC position was predicted via regression equations modified from the study by Harrington et al. [35]. The x-coordinate (craniocaudal direction) and z-coordinate (lateromedial direction) of the HJC were predicted with exclusive multivariate linear regression equations consisting of two independent variables and three parameters. The independent variables were pelvic width, as described by the distance between the bilateral iliac crest markers, and pelvic length, as determined by the distance between the midpoints of the bilateral iliac crest markers and the midpoints of the bilateral ischial tuberosity markers. The parameters of the regression equations were determined using 24 CT-derived surface pelvic models (12 Labrador retrievers and 12 mixed-breed dogs) from our previous studies and database [15, 36, 37]. The y-coordinate (proximodistal direction) of the HJC was determined as the negative distance between the GT marker and the posterior pelvic plane of the pelvis [37], similar to the method used in [9]. The SJC was predicted as the origin of the femoral AF. The 3-D coordinates of the joint centres were expressed in both AFs of adjacent body segments.

The aim of the MKO method was to estimate the 3-D poses of the pelvic limb by minimizing the sum of the squared distances between the model-determined and measured skin marker positions while being subjected to the kinematic constraints (Fig. 5). Here, the weighting factors can be applied to individual markers or segments to prioritize marker matching on different segments [22]. The model-determined marker positions were derived by spatially transforming the coordinates of the marker template based on the poses of the connected pelvic, femoral and tibial segment models expressed in 12 degree-of-freedoms (DOFs). Among them, 6 DOFs represented the translations and orientations of the pelvic segment in 3-D space, 3 DOFs represented femoral rotation about the HJC, and 3 DOFs represented tibial rotation about the SJC. The Levenberg-Marquardt algorithm was used to solve this nonlinear least squared problem [38]. The setup and customization of the multibody model, the prediction of joint centres and the kinematics estimation via the MKO were carried out using a self-developed application implemented in MATLAB (MATLAB R2017b, The Mathworks Inc., Natick, Massachusetts, USA).

### Study populations

The experimental protocol was approved by the National Taiwan University’s Institutional Animal Care and Use Committee. Eight client-owned skeletal mature Taiwan Dogs (age: 3.1 ± 1.2 years; body weight: 19.1 ± 3.8 kg; and BCS: 4.5/9) were recruited in the study. The Taiwan Dog is a medium-sized, non-chondrodystrophic breed that features a muscular and nearly square-shaped body and slender hindlimbs. All dogs underwent a thorough physical examination and radiographic examinations of the bilateral pelvic limbs, and found to be free of orthopedic abnormalities.

### Motion data collection

Kinematic data and fluoroscopic video were obtained from dogs walking on a treadmill (PetRun PR720F, Iwate International Developing Co., Ltd., Taichung, Taiwan) at a velocity of 0.7 m/s, using an integrated measurement unit. The integrated measurement unit consisted of an X-ray fluoroscopy (Arcadis Avantic, Siemens Healthineers, Munich, Germany) and a 9-camera motion capture system (Bonita B10 & Vero, Vicon Motion Systems Inc., Oxford, UK), as described in [15]. Fluoroscopy was performed in digital cine mode at a frame rate of 30 frames/s and a resolution of 1024 × 1024 pixels. The averaged tube voltage and tube current of the X-ray tube were 53 kVp and 27 mA, respectively. As a result, fluoroscopic images of the lateromedial view of the right stifle and 3-D trajectories of the skin markers on the pelvic limb were obtained. The marker data were acquired, pre-processed and semi-manually labelled using proprietary software (Nexus, Vicon Motion Systems Inc., Oxford, UK). At least three valid trials each with three complete gait cycles were collected, from which three gait cycles were extracted for subsequent analysis. The selection criteria for the gait cycles was that the tested stifle joint must be kept within the field of view of X-ray fluoroscopy during a complete gait cycle.

### Computed tomography acquisition

General anaesthesia was then induced in the dogs with propofol (4–6 mg/kg, Lipuro 1%, B. Braun Melsungen AG, Germany) and maintained using isoflurane (Attane, Panion & BF Biotech Inc., Taiwan). Each dog was positioned in ventral recumbency and received a CT scan (Activion 16, Toshiba Medical Systems Corporation, Tochigi, Japan) of the pelvic limbs, given a volumetric CT data set with a voxel size of 0.625 mm × 0.625 mm × 0.3 mm. After CT scanning, the dogs were recovered from anaesthesia and discharged under the care of their owners.

### Standard reference kinematics

The standard reference kinematics of the femur and tibia were obtained with CT model-based fluoroscopy using custom software developed by the authors [39, 40]. The workflow of the method is briefly described as follows. Volumetric bone models of the femur and tibia were extracted from the original CT data set. The spatial pose of the bone model was determined using a 3-D (bone model) to 2-D (fluoroscopy) image registration routine, in which the 3-D poses of the bone model were iteratively updated until the similarity between the X-ray fluoroscopic image and the simulated X-ray image (normally called digitally reconstructed radiograph) was maximized. The latter was generated by virtually projecting the volumetric bone model onto the image plane of the fluoroscopy [39]. STA-free pelvic poses were not available with the current fluoroscopic analysis because the pelvic bone was not in the field of view of the fluoroscopy, leading to the absence of the standard reference kinematic data of the pelvis. To address this issue, the pelvic poses could be determined but only with skin markers. Therefore, the hip joint angles calculated from the STA-free femoral poses and STA-affected pelvic poses cannot be recognized as completely STA-free.

The CT images were processed to isolate the femur regions after appropriate image segmentation, which were then used to reconstruct the polygonal mesh surface of the femur with the marching cube method [41]. CT image processing was executed using an in-house-developed application implemented in MATLAB. The reference SJC was determined as the centroid of the best-fitted cylinder to the point cloud of the bilateral condyles extracted from the CT-derived surface bone model [42]. The reference of the HJC location was taken as the centroid of the best-fitted sphere to the femoral head model.

### Evaluation of MKO and SO-determined kinematics

MKO was performed with the measured skin markers to reproduce the 3-D kinematics of the pelvic limb using the process as mentioned above. SO-determined kinematics were also obtained following previous methods [14, 18]. Joint kinematics were expressed in Cardan angles following a z-x-y rotation sequence, corresponding to flexion/extension, adduction/abduction and internal/external rotation [43].

The accuracy of the predicted joint centre locations and their influence on the MKO-determined kinematics were assessed by comparing them to reference values. The effects of weighting factors assigned to skin markers were also investigated. MKO with weightings specified by the reciprocal of “local marker displacement” was compared to that without imposing weightings. The local marker displacement was estimated by the averaged displacement amplitude of a marker from its mean position with respect to the underlying bone throughout a gait cycle.

### Statistics

The degree of agreement between MKO, and SO -determined joint kinematics and reference kinematics was evaluated via a coefficient of determination (R^2^) and bias and CI from Bland-Altman analysis [44]. Differences between MKO-determined and reference kinematics were computed, and for each subject, the RMSDs across three gait cycles were computed. The RMSDs resulting from the MKO-determined kinematics were compared to those obtained using SO-determined kinematics of the hip and stifle joints. The normality of the data being compared was tested using the Shapiro-Wilk test. Between-condition comparisons for variables passing the test were conducted using a paired t-test with a significance level of 0.05. Otherwise, the Wilcoxon signed-rank test was used. The statistical analyses were performed using MATLAB.

## Data Availability

The datasets used and/or analysed during the current study are available from the corresponding author on reasonable request.

## Abbreviations

2-D: Two-dimensional
3-D: Three-dimensional
AF: Anatomical frame
CI: Confidence interval
CT: Computed tomography
DOF: Degree-of-freedom
GT: Greater trochanter
HJC: Hip joint centre
MKO: Multibody kinematics optimization
RMSD: Root mean square difference
SJC: Stifle joint centre
SO: Single-body optimization
STA: Soft tissue artefact
